# Tricyclo­hexyl(2,3-dibromo-3-phenyl­propionato-κ*O*)tin(IV)

**DOI:** 10.1107/S1600536809012240

**Published:** 2009-04-08

**Authors:** Pui Yee Thong, Kong Mun Lo, Seik Weng Ng

**Affiliations:** a Department of Chemistry, University of Malaya, 50603 Kuala Lumpur, Malaysia

## Abstract

Tricyclo­hexyl­tin cinnamate reacts with 4,4-dimethyl­amino­pyridine hydro­bromide perbromide to form the title compound, [Sn(C_6_H_11_)_3_(C_9_H_7_Br_2_O_2_)], which exists as a monomeric mol­ecule with the Sn atom in a distorted tetra­hedral C_3_O coordination geometry.

## Related literature

For reviews of the structural chemistry of organotin carboxyl­ates, see: Tiekink (1991 ▶, 1994 ▶).
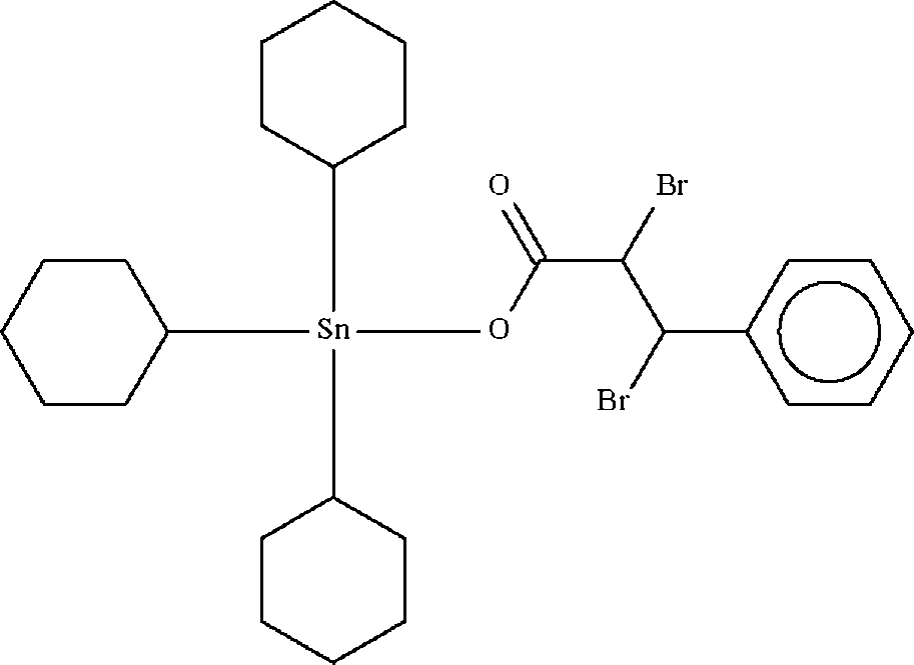


## Experimental

### 

#### Crystal data

[Sn(C_6_H_11_)_3_(C_9_H_7_Br_2_O_2_)]
*M* *_r_* = 675.10
Monoclinic, 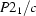

*a* = 21.2359 (3) Å
*b* = 9.0837 (1) Å
*c* = 15.0550 (2) Å
β = 108.287 (1)°
*V* = 2757.45 (6) Å^3^
*Z* = 4
Mo *K*α radiation
μ = 3.85 mm^−1^
*T* = 118 K
0.30 × 0.20 × 0.10 mm

#### Data collection

Bruker SMART APEX diffractometer
Absorption correction: multi-scan (*SADABS*; Sheldrick, 1996 ▶) *T*_min_ = 0.392, *T*_max_ = 0.700
21967 measured reflections
6325 independent reflections
5007 reflections with *I* > 2σ(*I*)
*R*_int_ = 0.034

#### Refinement

*R*[*F* ^2^ > 2σ(*F* ^2^)] = 0.032
*wR*(*F* ^2^) = 0.083
*S* = 1.04
6325 reflections
289 parameters
H-atom parameters constrained
Δρ_max_ = 1.63 e Å^−3^
Δρ_min_ = −1.13 e Å^−3^

### 

Data collection: *APEX2* (Bruker, 2008 ▶); cell refinement: *SAINT* (Bruker, 2008 ▶); data reduction: *SAINT*; program(s) used to solve structure: *SHELXS97* (Sheldrick, 2008 ▶); program(s) used to refine structure: *SHELXL97* (Sheldrick, 2008 ▶); molecular graphics: *X-SEED* (Barbour, 2001 ▶); software used to prepare material for publication: *publCIF* (Westrip, 2009 ▶).

## Supplementary Material

Crystal structure: contains datablocks global, I. DOI: 10.1107/S1600536809012240/tk2413sup1.cif

Structure factors: contains datablocks I. DOI: 10.1107/S1600536809012240/tk2413Isup2.hkl

Additional supplementary materials:  crystallographic information; 3D view; checkCIF report

## Figures and Tables

**Fig. 1.**
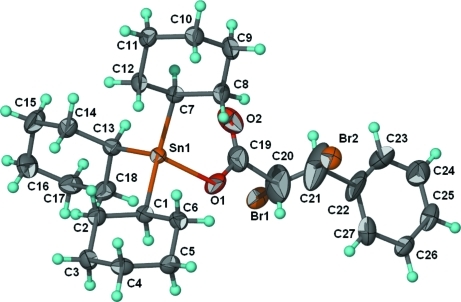
Thermal ellipsoid plot (Barbour, 2001) of Sn(C6H11)3(C9H7Br2O2) at the 70% probability level. Hydrogen atoms are drawn as spheres of arbitrary radius.

## supplementary crystallographic information

### Experimental

Tricyclohexyltin chloride (1.93 g, 5 mmol) was reacted with cinnamic acid (0.74 g, 5 mmol) in ethanol (100 ml) under reflux for 2 h. The product, tricyclohexyltin cinnamate, was collected upon removal of the solvent. The organotin compound (1 g, 2 mmol) and 4,4-dimethylaminopyridine hydrobromide perbromide (0.73 g, 2 mmol) were heated in 1:1 ethanol:chloroform (100 ml) for 1 h. Slow evaporation of the filtrate gave colorless crystals.

### Refinement

Carbon-bound H-atoms were placed in calculated positions (C–H 0.95 to 1.00 Å) and were included in the refinement in the riding model approximation, with *U*(H) set to 1.2*U*(C). The large peak/hole is in the vicinity (about 1 Å) of the C21 atom.

### Crystal data

**Table:**

[Sn(C_6_H_11_)_3_(C_9_H_7_Br_2_O_2_)]	*F*(000) = 1352
*M**_r_* = 675.10	*D*_x_ = 1.626 Mg m^−^^3^
Monoclinic, *P*2_1_/*c*	Mo *K*α radiation, λ = 0.71073 Å
Hall symbol: -P 2ybc	Cell parameters from 7301 reflections
*a* = 21.2359 (3) Å	θ = 2.5–28.2°
*b* = 9.0837 (1) Å	µ = 3.85 mm^−^^1^
*c* = 15.0550 (2) Å	*T* = 118 K
β = 108.287 (1)°	Block, colorless
*V* = 2757.45 (6) Å^3^	0.30 × 0.20 × 0.10 mm
*Z* = 4	

### Data collection

**Table:**

Bruker SMART APEX diffractometer	6325 independent reflections
Radiation source: fine-focus sealed tube	5007 reflections with *I* > 2σ(*I*)
graphite	*R*_int_ = 0.034
ω scans	θ_max_ = 27.5°, θ_min_ = 1.0°
Absorption correction: multi-scan (*SADABS*; Sheldrick, 1996)	*h* = −27→27
*T*_min_ = 0.392, *T*_max_ = 0.700	*k* = −11→11
21967 measured reflections	*l* = −18→19

### Refinement

**Table:**

Refinement on *F*^2^	Primary atom site location: structure-invariant direct methods
Least-squares matrix: full	Secondary atom site location: difference Fourier map
*R*[*F*^2^ > 2σ(*F*^2^)] = 0.032	Hydrogen site location: inferred from neighbouring sites
*wR*(*F*^2^) = 0.083	H-atom parameters constrained
*S* = 1.04	*w* = 1/[σ^2^(*F*_o_^2^) + (0.0318*P*)^2^ + 6.5266*P*] where *P* = (*F*_o_^2^ + 2*F*_c_^2^)/3
6325 reflections	(Δ/σ)_max_ = 0.001
289 parameters	Δρ_max_ = 1.63 e Å^−^^3^
0 restraints	Δρ_min_ = −1.13 e Å^−^^3^

### Fractional atomic coordinates and isotropic or equivalent isotropic displacement parameters (Å^2^)

**Table:**

	*x*	*y*	*z*	*U*_iso_*/*U*_eq_	
Sn1	0.213693 (12)	0.54260 (3)	0.484115 (16)	0.02051 (7)	
Br1	0.43779 (2)	0.37225 (5)	0.44008 (3)	0.03484 (11)	
Br2	0.258387 (19)	0.05999 (4)	0.32990 (3)	0.03129 (10)	
O1	0.26589 (15)	0.4358 (3)	0.4077 (2)	0.0387 (7)	
O2	0.31430 (15)	0.2973 (3)	0.5299 (2)	0.0452 (8)	
C1	0.15692 (17)	0.6666 (4)	0.3632 (2)	0.0220 (7)	
H1	0.1895	0.7061	0.3336	0.026*	
C2	0.12163 (19)	0.7996 (4)	0.3873 (3)	0.0264 (8)	
H2A	0.0894	0.7659	0.4183	0.032*	
H2B	0.1545	0.8636	0.4318	0.032*	
C3	0.08542 (19)	0.8880 (4)	0.3002 (3)	0.0279 (8)	
H3A	0.0604	0.9690	0.3177	0.033*	
H3B	0.1183	0.9324	0.2739	0.033*	
C4	0.03775 (18)	0.7926 (4)	0.2262 (3)	0.0275 (8)	
H4A	0.0178	0.8519	0.1691	0.033*	
H4B	0.0015	0.7587	0.2494	0.033*	
C5	0.07263 (19)	0.6598 (4)	0.2018 (2)	0.0282 (8)	
H5A	0.1051	0.6933	0.1711	0.034*	
H5B	0.0396	0.5964	0.1571	0.034*	
C6	0.1087 (2)	0.5702 (4)	0.2895 (3)	0.0276 (8)	
H6A	0.0757	0.5270	0.3161	0.033*	
H6B	0.1333	0.4885	0.2721	0.033*	
C7	0.16265 (18)	0.3824 (4)	0.5421 (2)	0.0232 (7)	
H7	0.1946	0.3502	0.6033	0.028*	
C8	0.1404 (2)	0.2437 (4)	0.4836 (3)	0.0293 (8)	
H8A	0.1093	0.2709	0.4217	0.035*	
H8B	0.1794	0.1954	0.4735	0.035*	
C9	0.1064 (2)	0.1361 (4)	0.5322 (3)	0.0338 (9)	
H9A	0.0899	0.0502	0.4909	0.041*	
H9B	0.1392	0.1000	0.5905	0.041*	
C10	0.04875 (19)	0.2078 (4)	0.5554 (3)	0.0289 (8)	
H10A	0.0300	0.1371	0.5903	0.035*	
H10B	0.0135	0.2334	0.4967	0.035*	
C11	0.0710 (2)	0.3462 (4)	0.6139 (3)	0.0307 (9)	
H11A	0.1028	0.3193	0.6754	0.037*	
H11B	0.0322	0.3939	0.6248	0.037*	
C12	0.10404 (19)	0.4543 (4)	0.5642 (3)	0.0265 (8)	
H12A	0.0711	0.4879	0.5053	0.032*	
H12B	0.1198	0.5415	0.6044	0.032*	
C13	0.28327 (18)	0.6755 (4)	0.5863 (2)	0.0229 (7)	
H13	0.3158	0.6080	0.6299	0.028*	
C14	0.2482 (2)	0.7634 (4)	0.6445 (3)	0.0302 (8)	
H14A	0.2135	0.8265	0.6024	0.036*	
H14B	0.2263	0.6943	0.6764	0.036*	
C15	0.2972 (2)	0.8594 (5)	0.7173 (3)	0.0399 (10)	
H15A	0.2729	0.9192	0.7509	0.048*	
H15B	0.3290	0.7957	0.7637	0.048*	
C16	0.3352 (2)	0.9610 (5)	0.6714 (3)	0.0399 (10)	
H16A	0.3039	1.0314	0.6299	0.048*	
H16B	0.3682	1.0182	0.7203	0.048*	
C17	0.3701 (2)	0.8748 (5)	0.6152 (3)	0.0350 (9)	
H17A	0.4047	0.8121	0.6579	0.042*	
H17B	0.3923	0.9440	0.5839	0.042*	
C18	0.3220 (2)	0.7777 (5)	0.5415 (3)	0.0333 (9)	
H18A	0.3472	0.7180	0.5092	0.040*	
H18B	0.2906	0.8407	0.4944	0.040*	
C19	0.3064 (2)	0.3375 (5)	0.4512 (4)	0.0428 (11)	
C20	0.3465 (4)	0.2732 (7)	0.3902 (6)	0.086 (2)	
H20	0.3254	0.3114	0.3254	0.103*	
C21	0.3511 (4)	0.1324 (10)	0.3833 (6)	0.103 (3)	
H21	0.3661	0.0957	0.4493	0.123*	
C22	0.3980 (3)	0.0619 (6)	0.3377 (4)	0.0589 (15)	
C23	0.4391 (3)	−0.0514 (6)	0.3837 (4)	0.0600 (15)	
H23	0.4393	−0.0795	0.4445	0.072*	
C24	0.4791 (3)	−0.1224 (6)	0.3434 (3)	0.0489 (12)	
H24	0.5066	−0.2006	0.3757	0.059*	
C25	0.4800 (2)	−0.0820 (5)	0.2563 (3)	0.0362 (10)	
H25	0.5080	−0.1328	0.2282	0.043*	
C26	0.4408 (2)	0.0312 (5)	0.2089 (3)	0.0345 (9)	
H26	0.4420	0.0589	0.1486	0.041*	
C27	0.3996 (2)	0.1048 (5)	0.2490 (3)	0.0436 (11)	
H27	0.3727	0.1837	0.2167	0.052*	

### Atomic displacement parameters (Å^2^)

**Table:**

	*U*^11^	*U*^22^	*U*^33^	*U*^12^	*U*^13^	*U*^23^
Sn1	0.02214 (13)	0.02028 (12)	0.02122 (12)	0.00165 (10)	0.00982 (9)	0.00100 (10)
Br1	0.0356 (2)	0.0413 (2)	0.0301 (2)	−0.01657 (18)	0.01398 (17)	−0.01001 (18)
Br2	0.0250 (2)	0.0332 (2)	0.0360 (2)	−0.00672 (16)	0.01009 (16)	−0.00529 (17)
O1	0.0474 (18)	0.0441 (18)	0.0341 (16)	0.0140 (15)	0.0263 (14)	0.0022 (14)
O2	0.0359 (17)	0.0354 (17)	0.062 (2)	0.0099 (14)	0.0122 (15)	0.0110 (16)
C1	0.0203 (17)	0.0223 (18)	0.0237 (18)	0.0007 (14)	0.0074 (14)	0.0011 (14)
C2	0.028 (2)	0.0254 (19)	0.0253 (19)	0.0027 (16)	0.0082 (15)	−0.0006 (15)
C3	0.031 (2)	0.0236 (19)	0.028 (2)	0.0009 (16)	0.0076 (16)	−0.0007 (16)
C4	0.0256 (19)	0.032 (2)	0.0227 (18)	−0.0020 (16)	0.0040 (15)	0.0050 (16)
C5	0.032 (2)	0.029 (2)	0.0205 (18)	−0.0075 (16)	0.0043 (15)	−0.0031 (15)
C6	0.035 (2)	0.0218 (19)	0.0246 (19)	−0.0027 (16)	0.0071 (16)	−0.0005 (15)
C7	0.0257 (19)	0.0220 (18)	0.0228 (18)	−0.0010 (15)	0.0089 (15)	0.0011 (14)
C8	0.034 (2)	0.0249 (19)	0.034 (2)	−0.0017 (16)	0.0180 (17)	−0.0064 (16)
C9	0.042 (2)	0.0209 (19)	0.045 (2)	−0.0004 (17)	0.023 (2)	−0.0037 (17)
C10	0.032 (2)	0.0251 (19)	0.033 (2)	−0.0035 (16)	0.0154 (17)	−0.0015 (16)
C11	0.038 (2)	0.028 (2)	0.034 (2)	−0.0040 (17)	0.0220 (18)	−0.0049 (17)
C12	0.031 (2)	0.0194 (18)	0.035 (2)	−0.0029 (16)	0.0189 (17)	−0.0048 (16)
C13	0.0227 (18)	0.0240 (18)	0.0206 (17)	0.0039 (15)	0.0047 (14)	0.0033 (14)
C14	0.033 (2)	0.032 (2)	0.029 (2)	−0.0006 (17)	0.0143 (17)	−0.0025 (17)
C15	0.049 (3)	0.043 (3)	0.032 (2)	−0.010 (2)	0.019 (2)	−0.013 (2)
C16	0.047 (3)	0.030 (2)	0.042 (2)	−0.010 (2)	0.012 (2)	−0.0065 (19)
C17	0.037 (2)	0.036 (2)	0.032 (2)	−0.0117 (19)	0.0109 (18)	0.0017 (18)
C18	0.035 (2)	0.039 (2)	0.029 (2)	−0.0110 (18)	0.0153 (17)	−0.0035 (18)
C19	0.043 (3)	0.036 (2)	0.058 (3)	0.008 (2)	0.028 (2)	−0.002 (2)
C20	0.089 (5)	0.067 (4)	0.129 (6)	0.024 (4)	0.073 (5)	−0.014 (4)
C21	0.088 (5)	0.115 (6)	0.129 (7)	−0.017 (5)	0.069 (5)	−0.069 (6)
C22	0.073 (4)	0.064 (4)	0.059 (3)	−0.002 (3)	0.048 (3)	−0.026 (3)
C23	0.096 (5)	0.057 (3)	0.039 (3)	−0.001 (3)	0.039 (3)	−0.004 (3)
C24	0.058 (3)	0.049 (3)	0.036 (3)	0.005 (2)	0.008 (2)	−0.004 (2)
C25	0.028 (2)	0.043 (2)	0.041 (2)	−0.0025 (18)	0.0155 (18)	−0.017 (2)
C26	0.045 (2)	0.035 (2)	0.030 (2)	−0.0115 (19)	0.0210 (19)	−0.0097 (18)
C27	0.048 (3)	0.033 (2)	0.050 (3)	0.006 (2)	0.016 (2)	−0.012 (2)

### Geometric parameters (Å, °)

**Table:**

Sn1—O1	2.072 (3)	C11—H11A	0.9900
Sn1—C13	2.141 (4)	C11—H11B	0.9900
Sn1—C7	2.157 (3)	C12—H12A	0.9900
Sn1—C1	2.158 (3)	C12—H12B	0.9900
Br1—C20	2.054 (8)	C13—C18	1.530 (5)
Br2—C21	1.990 (7)	C13—C14	1.539 (5)
O1—C19	1.271 (5)	C13—H13	1.0000
O2—C19	1.201 (6)	C14—C15	1.526 (6)
C1—C6	1.528 (5)	C14—H14A	0.9900
C1—C2	1.525 (5)	C14—H14B	0.9900
C1—H1	1.0000	C15—C16	1.526 (6)
C2—C3	1.525 (5)	C15—H15A	0.9900
C2—H2A	0.9900	C15—H15B	0.9900
C2—H2B	0.9900	C16—C17	1.507 (6)
C3—C4	1.520 (5)	C16—H16A	0.9900
C3—H3A	0.9900	C16—H16B	0.9900
C3—H3B	0.9900	C17—C18	1.530 (6)
C4—C5	1.519 (5)	C17—H17A	0.9900
C4—H4A	0.9900	C17—H17B	0.9900
C4—H4B	0.9900	C18—H18A	0.9900
C5—C6	1.536 (5)	C18—H18B	0.9900
C5—H5A	0.9900	C19—C20	1.548 (7)
C5—H5B	0.9900	C20—C21	1.290 (10)
C6—H6A	0.9900	C20—H20	1.0000
C6—H6B	0.9900	C21—C22	1.517 (7)
C7—C12	1.531 (5)	C21—H21	1.0000
C7—C8	1.525 (5)	C22—C23	1.387 (8)
C7—H7	1.0000	C22—C27	1.402 (7)
C8—C9	1.531 (5)	C23—C24	1.351 (7)
C8—H8A	0.9900	C23—H23	0.9500
C8—H8B	0.9900	C24—C25	1.369 (6)
C9—C10	1.521 (5)	C24—H24	0.9500
C9—H9A	0.9900	C25—C26	1.374 (6)
C9—H9B	0.9900	C25—H25	0.9500
C10—C11	1.523 (5)	C26—C27	1.381 (6)
C10—H10A	0.9900	C26—H26	0.9500
C10—H10B	0.9900	C27—H27	0.9500
C11—C12	1.531 (5)		
			
O1—Sn1—C13	107.13 (13)	C7—C12—H12A	109.4
O1—Sn1—C7	109.48 (13)	C11—C12—H12A	109.4
C13—Sn1—C7	114.25 (13)	C7—C12—H12B	109.4
O1—Sn1—C1	91.76 (12)	C11—C12—H12B	109.4
C13—Sn1—C1	112.88 (13)	H12A—C12—H12B	108.0
C7—Sn1—C1	118.36 (14)	C18—C13—C14	111.1 (3)
C19—O1—Sn1	115.9 (3)	C18—C13—Sn1	111.7 (2)
C6—C1—C2	111.1 (3)	C14—C13—Sn1	110.7 (2)
C6—C1—Sn1	112.5 (2)	C18—C13—H13	107.7
C2—C1—Sn1	113.5 (2)	C14—C13—H13	107.7
C6—C1—H1	106.4	Sn1—C13—H13	107.7
C2—C1—H1	106.4	C15—C14—C13	111.2 (3)
Sn1—C1—H1	106.4	C15—C14—H14A	109.4
C3—C2—C1	111.4 (3)	C13—C14—H14A	109.4
C3—C2—H2A	109.3	C15—C14—H14B	109.4
C1—C2—H2A	109.3	C13—C14—H14B	109.4
C3—C2—H2B	109.3	H14A—C14—H14B	108.0
C1—C2—H2B	109.3	C16—C15—C14	111.0 (3)
H2A—C2—H2B	108.0	C16—C15—H15A	109.4
C4—C3—C2	111.7 (3)	C14—C15—H15A	109.4
C4—C3—H3A	109.3	C16—C15—H15B	109.4
C2—C3—H3A	109.3	C14—C15—H15B	109.4
C4—C3—H3B	109.3	H15A—C15—H15B	108.0
C2—C3—H3B	109.3	C17—C16—C15	111.2 (4)
H3A—C3—H3B	107.9	C17—C16—H16A	109.4
C3—C4—C5	111.5 (3)	C15—C16—H16A	109.4
C3—C4—H4A	109.3	C17—C16—H16B	109.4
C5—C4—H4A	109.3	C15—C16—H16B	109.4
C3—C4—H4B	109.3	H16A—C16—H16B	108.0
C5—C4—H4B	109.3	C16—C17—C18	111.9 (4)
H4A—C4—H4B	108.0	C16—C17—H17A	109.2
C4—C5—C6	111.3 (3)	C18—C17—H17A	109.2
C4—C5—H5A	109.4	C16—C17—H17B	109.2
C6—C5—H5A	109.4	C18—C17—H17B	109.2
C4—C5—H5B	109.4	H17A—C17—H17B	107.9
C6—C5—H5B	109.4	C17—C18—C13	111.0 (3)
H5A—C5—H5B	108.0	C17—C18—H18A	109.4
C1—C6—C5	111.1 (3)	C13—C18—H18A	109.4
C1—C6—H6A	109.4	C17—C18—H18B	109.4
C5—C6—H6A	109.4	C13—C18—H18B	109.4
C1—C6—H6B	109.4	H18A—C18—H18B	108.0
C5—C6—H6B	109.4	O2—C19—O1	125.8 (4)
H6A—C6—H6B	108.0	O2—C19—C20	122.6 (5)
C12—C7—C8	110.6 (3)	O1—C19—C20	111.6 (5)
C12—C7—Sn1	110.2 (2)	C21—C20—C19	119.5 (6)
C8—C7—Sn1	115.3 (2)	C21—C20—Br1	112.1 (6)
C12—C7—H7	106.7	C19—C20—Br1	104.4 (4)
C8—C7—H7	106.7	C21—C20—H20	106.7
Sn1—C7—H7	106.7	C19—C20—H20	106.7
C9—C8—C7	111.0 (3)	Br1—C20—H20	106.7
C9—C8—H8A	109.4	C20—C21—C22	122.2 (7)
C7—C8—H8A	109.4	C20—C21—Br2	105.6 (6)
C9—C8—H8B	109.4	C22—C21—Br2	112.6 (4)
C7—C8—H8B	109.4	C20—C21—H21	105.0
H8A—C8—H8B	108.0	C22—C21—H21	105.0
C10—C9—C8	111.8 (3)	Br2—C21—H21	105.0
C10—C9—H9A	109.3	C23—C22—C27	118.8 (4)
C8—C9—H9A	109.3	C23—C22—C21	119.1 (6)
C10—C9—H9B	109.3	C27—C22—C21	122.0 (6)
C8—C9—H9B	109.3	C24—C23—C22	121.1 (5)
H9A—C9—H9B	107.9	C24—C23—H23	119.5
C9—C10—C11	111.1 (3)	C22—C23—H23	119.5
C9—C10—H10A	109.4	C23—C24—C25	120.1 (5)
C11—C10—H10A	109.4	C23—C24—H24	119.9
C9—C10—H10B	109.4	C25—C24—H24	119.9
C11—C10—H10B	109.4	C24—C25—C26	120.6 (4)
H10A—C10—H10B	108.0	C24—C25—H25	119.7
C10—C11—C12	110.7 (3)	C26—C25—H25	119.7
C10—C11—H11A	109.5	C25—C26—C27	120.0 (4)
C12—C11—H11A	109.5	C25—C26—H26	120.0
C10—C11—H11B	109.5	C27—C26—H26	120.0
C12—C11—H11B	109.5	C26—C27—C22	119.4 (5)
H11A—C11—H11B	108.1	C26—C27—H27	120.3
C7—C12—C11	111.1 (3)	C22—C27—H27	120.3
			
C13—Sn1—O1—C19	69.2 (3)	O1—Sn1—C13—C14	178.1 (2)
C7—Sn1—O1—C19	−55.2 (3)	C7—Sn1—C13—C14	−60.5 (3)
C1—Sn1—O1—C19	−176.2 (3)	C1—Sn1—C13—C14	78.6 (3)
O1—Sn1—C1—C6	69.1 (3)	C18—C13—C14—C15	−54.9 (4)
C13—Sn1—C1—C6	178.5 (2)	Sn1—C13—C14—C15	−179.7 (3)
C7—Sn1—C1—C6	−44.2 (3)	C13—C14—C15—C16	55.5 (5)
O1—Sn1—C1—C2	−163.7 (3)	C14—C15—C16—C17	−56.1 (5)
C13—Sn1—C1—C2	−54.2 (3)	C15—C16—C17—C18	56.1 (5)
C7—Sn1—C1—C2	83.0 (3)	C16—C17—C18—C13	−55.3 (5)
C6—C1—C2—C3	−55.1 (4)	C14—C13—C18—C17	54.2 (4)
Sn1—C1—C2—C3	176.9 (2)	Sn1—C13—C18—C17	178.4 (3)
C1—C2—C3—C4	54.8 (4)	Sn1—O1—C19—O2	3.9 (7)
C2—C3—C4—C5	−54.7 (4)	Sn1—O1—C19—C20	−175.7 (4)
C3—C4—C5—C6	54.9 (4)	O2—C19—C20—C21	49.4 (11)
C2—C1—C6—C5	55.4 (4)	O1—C19—C20—C21	−131.0 (8)
Sn1—C1—C6—C5	−176.1 (2)	O2—C19—C20—Br1	−76.9 (6)
C4—C5—C6—C1	−55.3 (4)	O1—C19—C20—Br1	102.7 (5)
O1—Sn1—C7—C12	−157.5 (2)	C19—C20—C21—C22	−170.2 (6)
C13—Sn1—C7—C12	82.4 (3)	Br1—C20—C21—C22	−47.6 (11)
C1—Sn1—C7—C12	−54.3 (3)	C19—C20—C21—Br2	59.5 (9)
O1—Sn1—C7—C8	−31.4 (3)	Br1—C20—C21—Br2	−177.9 (3)
C13—Sn1—C7—C8	−151.5 (3)	C20—C21—C22—C23	131.4 (9)
C1—Sn1—C7—C8	71.8 (3)	Br2—C21—C22—C23	−101.3 (7)
C12—C7—C8—C9	−55.5 (4)	C20—C21—C22—C27	−50.6 (11)
Sn1—C7—C8—C9	178.6 (3)	Br2—C21—C22—C27	76.7 (7)
C7—C8—C9—C10	55.3 (5)	C27—C22—C23—C24	−1.7 (9)
C8—C9—C10—C11	−55.4 (5)	C21—C22—C23—C24	176.4 (6)
C9—C10—C11—C12	55.8 (5)	C22—C23—C24—C25	0.7 (9)
C8—C7—C12—C11	56.6 (4)	C23—C24—C25—C26	0.4 (8)
Sn1—C7—C12—C11	−174.7 (3)	C24—C25—C26—C27	−0.5 (7)
C10—C11—C12—C7	−56.7 (4)	C25—C26—C27—C22	−0.5 (7)
O1—Sn1—C13—C18	53.7 (3)	C23—C22—C27—C26	1.5 (8)
C7—Sn1—C13—C18	175.1 (3)	C21—C22—C27—C26	−176.5 (5)
C1—Sn1—C13—C18	−45.8 (3)		
